# Better compliance with hypofractionation vs. conventional fractionation in adjuvant breast cancer radiotherapy: Results of a single, institutional, retrospective study

**DOI:** 10.1007/s00066-017-1115-z

**Published:** 2017-02-23

**Authors:** Volker Rudat, Alaa Nour, Mohamed Hammoud, Salam Abou Ghaida

**Affiliations:** Department of Radiation Oncology, Saad Specialist Hospital, 31952 Al Khobar, Saudi Arabia

**Keywords:** Breast neoplasms, Radiotherapy, Dose hypofractionation, Radiation injuries, Risk factors, Neoplasien der Mamma, Strahlentherapie, Hypofraktionierung, Strahlenbedingte Nebenwirkungen, Risikofaktoren

## Abstract

**Background:**

The aim of the study was to identify factors significantly associated with the occurrence of unintended treatment interruptions in adjuvant breast cancer radiotherapy.

**Patients and methods:**

Patients treated with postoperative radiotherapy of the breast or chest wall between March 2014 and August 2016 were evaluated. The radiotherapy regimens and techniques applied were either conventional fractionation (CF; 28 daily fractions of 1.8 Gy or 25 fractions of 2.0 Gy) or hypofractionation (HF; 15 daily fractions of 2.67 Gy) with inverse planned intensity-modulated radiotherapy (IMRT) or three-dimensional planned conformal radiotherapy (3DCRT). Logistic regression analysis was used to identify factors associated with noncompliance. Noncompliance was defined as the missing of at least one scheduled radiotherapy fraction.

**Results:**

In all, 19 of 140 (13.6%) patients treated with HF and 39 of 146 (26.7%) treated with CF experienced treatment interruptions. Of 23 factors tested, the fractionation regimen emerged as the only independent significant prognostic factor for noncompliance on multivariate analysis (CF; *p* = 0.007; odds ratio, 2.3; 95% confidence interval, 1.3–4.2). No statistically significant differences concerning the reasons for treatment interruptions could be detected between patients treated with CF or HF.

**Conclusion:**

HF is significantly associated with a better patient compliance with the prescribed radiotherapy schedule compared with CF. The data suggest that this finding is basically related to the shorter overall treatment time of HF.

## Zusammenfassung

**Hintergrund:**

Ziel der Untersuchung war es, Faktoren zu identifizieren, die mit ungeplanten Behandlungsunterbrechungen bei der adjuvanten Strahlentherapie des Mammakarzinoms assoziiert sind.

**Methoden und Patienten:**

Es wurden Patienten untersucht, die eine adjuvante Strahlentherapie der Mamma oder Brustwand zwischen März 2014 und August 2016 erhielten. Zur Anwendung kamen als Fraktionierungsprotokoll und strahlentherapeutische Technik eine konventionell fraktionierte (CF; 28 Fraktionen mit 1,80 Gy oder 25 Fraktionen mit 2,00 Gy) oder eine hypofraktionierte Strahlentherapie (HF; 15 Fraktionen mit 2,67 Gy), eine intensitätsmodulierte (IMRT) oder dreidimensional geplante konformale Strahlentherapie (3DCRT). Mögliche mit Noncompliance assoziierte Faktoren wurden mittels logistischer Regressionsanalyse untersucht. Noncompliance wurde als gegeben betrachtet bei dem Ausfall mindestens einer geplanten Strahlentherapiefraktion.

**Ergebnisse:**

Bei 19 von 140 (13,6 %) Patienten mit HF und 39 von 146 (26,7 %), die mit CF behandelt wurden, traten ungeplante Behandlungsunterbrechungen auf. Von 23 untersuchten Faktoren ging als einziger unabhängiger signifikanter Faktor für Noncompliance das Fraktionierungsprotokoll aus der multivariaten Analyse hervor (CF; *p* = 0,007; Odds Ratio: 2,3; 95%-Konfidenzintervall: 1,3–4,2). Bezüglich der Ursachen ungeplanter Behandlungsunterbrechungen konnte kein statistisch signifikanter Unterschied festgestellt werden.

**Schlussfolgerung:**

Die HF ist signifikant mit einer besseren Patientencompliance im Vergleich zur CF assoziiert. Die Daten legen nahe, dass diese Assoziation hauptsächlich auf die kürzere Gesamtbehandlungszeit der HF zurückzuführen ist.

## Background

Unintended treatment interruptions may lead to a prolongation of the prescribed overall treatment time. For radiotherapy with curative intent, prolongation of the prescribed overall treatment time has been linked to inferior clinical outcomes [1–3]. This association appears to be consistent across many disease sites including head and neck cancer, cervical cancer, lung cancer, breast cancer, and other cancers [4, 5]. Prospective and retrospective studies have shown that treatment prolongation can increase the risk of local recurrence by up to 2% per day for certain malignancies [5].

The association between prolongation of the prescribed overall treatment time and inferior clinical outcomes has been explained with an accelerated repopulation of tumor clonogens, which can occur after treatment initiation [6]. It has also been reported that noncompliance may serve as a behavioral biomarker for other risk factors that contribute to poor outcomes, such as noncompliance with other important clinician visits and procedures, lack of social support, and mood disorders [4].

In this study, we analyzed the compliance to the prescribed radiotherapy schedule of breast cancer patients treated with postoperative radiotherapy of the whole breast or chest wall. The goal of the study was to identify factors significantly associated with the occurrence of treatment interruptions.

## Patients and methods

### Data collection and patient selection

The electronic patient files of 286 consecutive unselected patients treated with adjuvant breast cancer radiotherapy between March 2014 and August 2016 were reviewed. Eligibility criteria for the analysis were (a) histologically proven diagnosis of breast cancer or carcinoma in situ and (b) treatment with adjuvant postoperative radiotherapy after breast-conserving surgery or mastectomy. Exclusion criteria were bilateral breast cancer or history of previous radiotherapy of the breast or chest wall.

Patients were treated with either conventional fractionation (CF; 28 daily fractions of 1.8 Gy or 25 fractions of 2.0 Gy) or hypofractionation (HF; 15 daily fractions of 2.67 Gy). Where indicated, an electron boost was applied (five or eight daily fractions of 2.0 Gy). Radiotherapy fractions were scheduled once per day and five times per week. Patients who missed radiotherapy fractions were offered to be treated on weekends in order not to exceed the prescribed overall treatment time. The radiation techniques used were inverse planned intensity-modulated radiotherapy (IMRT) or three-dimensional planned conformal radiotherapy using wedge compensation (3DCRT). The patients were thoroughly informed about the pros and cons of the two fractionation regimens and radiation techniques, and the treatment decision was mainly based on patient preference. Patients not covered, or not fully covered, by medical insurance tended to opt for 3DCRT for financial reasons. Patients with personal commitments limiting the overall treatment time or patients living far away from the radiotherapy facility tended to opt for HF.

The acute radiation reactions and reasons for treatment interruptions were documented prospectively in the Local Area Network Therapy Information System “Lantis” (Siemens Healthcare, Germany). The acute radiation reactions were assessed once weekly during radiotherapy and 6 weeks after radiotherapy by two observers using the Common Terminology Criteria for Adverse Events (CTCAE v4.03). The two observers were not involved in the statistical analysis of the study, and a table with all weekly assessments was included in the “End of Treatment Report” of all patients. The maximum acute radiation reaction observed during the full course of the radiotherapy (including the boost to the tumor bed if applied) was used for the statistical analysis. Treatment interruptions were defined as missing at least one of the scheduled daily radiotherapy fractions. The reasons for treatment interruptions were categorized into “public holidays,” “patient unwillingness,” “machine breakdown,” “radiation reactions,” and “unspecified,” and documented prospectively together with the length of the treatment interruption.

The study was approved by the local institutional ethics committee and conducted in accordance with the Helsinki Declaration in its current version.

### Treatment planning and radiation techniques

The treatment planning and radiation techniques used for this study have been described in detail elsewhere [7–9]. In short, a non-contrast computed tomography (CT) simulation with a slice thickness of 5 mm was performed with the patient in the supine position. The planning target volume (PTV) of the whole breast or chest wall was defined according to the recommendations of the breast cancer atlas for radiation therapy planning consensus definitions of the Radiation Therapy Oncology Group (RTOG) [10]. The IMRT and 3DCRT plans were generated using the treatment planning system XIO 4.4 (CMS, Inc., St. Louis, Mo.). The dose to the PTV was prescribed according to the International Commission on Radiation Units and Measurement (ICRU) Reports 50 and 62 recommendations. Two Siemens Oncor Anvantgarde linear accelerators with a 160 MLC Multileaf Collimator were used for the treatment. Daily online verification and correction of the patient positioning error prior to radiotherapy were performed for all patients using orthogonal megavoltage electronic portal images [11]. No respiratory gating [12–14], integrated boost [15, 16], or partial breast irradiation [17] techniques were applied in this study. Two tangential semi-opposed beams, physical wedges (usually 15° or 30°), a 160 MLC Multileaf Collimator and 6 MV photons were used for the IMRT and 3DCRT plans. Occasionally a mixed-beam technique using 6 MV and 15 MV photons was used for the 3DCRT plans. Inverse treatment planning and a step-and-shoot technique were used for all IMRT plans. Tissue inhomogeneities were considered in the treatment planning optimization process, and the dose calculation algorithm used was “Superposition.” A few patients with left-sided breast cancer and unfavorable thoracic geometry were treated with seven-field IMRT in order to reduce the high-dose region to the heart [18].

### Statistical analysis

Differences between patient groups stratified by the occurrence of treatment interruptions (Table 1) or by the fractionation regimen (Table 3) were assessed using the chi-square test or *t* test where appropriate. To assess the association of multiple factors with the occurrence of treatment interruptions, a univariate and multivariate logistic regression analysis was performed. The factors tested in the logistic regression analysis are listed in Table 2. The model selection of the multivariate analysis was performed by a backward stepwise strategy. All tests were two-sided, and a *p* value of ≤0.05 was considered significant.

## Results

In total, 58 of 286 (20.3%) patients experienced treatment interruptions. The patient, disease, and treatment characteristics of the study population stratified by the occurrence of treatment interruptions are demonstrated in Table 1. As expected, the mean age of the study population was considerably lower compared with reports from Europe or the United States, most likely due to the young age structure of the general population [19].

**Table 1:** Patient, disease, and treatment characteristics stratified by occurrence of treatment interruptions

Characteristics		Total		Treatment interruptions				*p*
				Yes		No		
		*n*	%	*n*	%	*n*	%	
Patients		286	100	58	20.3	228	79.7	–
Country of origin	Middle East	208	72.7	42	72.4	166	72.8	0.18
	Asia	42	14.7	7	12.1	35	15.4	–
	Africa	25	8.7	4	6.9	21	9.2	–
	Europe/USA	11	3.8	5	8.6	6	2.6	–
Age at diagnosis (years)	Mean (SD)	48	(9.6)	48	(8.9)	49	(9.8)	0.65^a^
Body mass index	<25	47	16.5	12	20.7	35	15.4	0.57
	25–29	76	26.7	16	27.6	60	26.4	–
	≥30	162	56.8	30	51.7	132	58.1	–
Menopausal status	Premenopausal	143	50.0	31	53.4	112	49.1	0.56
	Postmenopausal	143	50.0	27	46.6	116	50.9	–
Marital status	Married	270	94.4	57	98.3	213	93.4	0.15
	Single	16	5.6	1	1.7	15	6.6	–
Financial status	Medical insurance	163	57.0	30	51.7	133	58.3	0.36
	Cash	123	43.0	28	48.3	95	41.7	–
Distance from home to treatment facility (km)	≤50	183	64.0	39	67.2	144	63.2	0.65
	51–100	62	21.7	10	17.2	52	22.8	–
	>100	41	14.3	9	15.5	32	14.0	–
Pathohistology	Invasive ductal cancer	264	92.3	53	91.4	211	92.5	0.83
	Invasive lobular cancer	15	5.2	4	6.9	11	4.8	–
	DCIS	5	1.7	1	1.7	4	1.8	–
	Other	2	0.7	0	0.0	2	0.9	–
Grading	G1	22	7.7	6	10.3	16	7.0	0.31
	G2	91	31.8	20	34.5	71	31.1	–
	G3	146	51.0	24	41.4	122	53.5	–
	Not reported	27	9.4	8	13.8	19	8.3	–
T classification	pTis	6	2.1	2	3.4	4	1.8	0.92
	pT0	8	2.8	2	3.4	6	2.6	–
	pT1	98	34.3	22	37.9	76	33.3	–
	pT2	114	39.9	22	37.9	92	40.4	–
	pT3	30	10.5	4	6.9	26	11.4	–
	pT4	20	7.0	4	6.9	16	7.0	–
	Not reported	10	3.5	2	3.4	8	3.5	–
N classification	pN0	102	35.7	28	48.3	74	32.5	0.20
	pN1	84	29.4	13	22.4	71	31.1	–
	pN2	60	21.0	12	20.7	48	21.1	–
	pN3	33	11.5	4	6.9	29	12.7	–
	Not reported	7	2.4	1	1.7	6	2.6	–
M classification	cM0	282	98.6	58	100.0	224	98.2	0.31
	cM1	4	1.4	0	0.0	4	1.8	–
ER status	Positive	204	71.3	43	74.1	161	70.6	0.62
	Negative	71	24.8	14	24.1	57	25.0	–
	Not reported	11	3.8	1	1.7	10	4.4	–
PR status	Positive	184	64.3	37	63.8	147	64.5	0.89
	Negative	86	30.1	17	29.3	69	30.3	–
	Not reported	16	5.6	4	6.9	12	5.3	–
Her2/neu status	Positive	79	27.6	17	29.3	62	27.2	0.94
	Negative	188	65.7	37	63.8	151	66.2	–
	Not reported	19	6.6	4	6.9	15	6.6	–
Planning target volume (PTV)	Chest wall	150	52.4	27	46.6	123	53.9	0.31
	Whole breast	136	47.6	31	53.4	105	46.1	–
Volume of PTV (cm^3^)	≤652	71	24.8	9	15.5	62	27.2	0.33
	653–872	72	25.2	17	29.3	55	24.1	–
	873–1235	71	24.8	16	27.6	55	24.1	–
	≥1236	72	25.2	16	27.6	56	24.6	–
Locoregional lymph nodes treated as part of plan	Yes	149	52.1	25	43.1	124	54.4	0.12
	No	137	47.9	33	56.9	104	45.6	–
Boost to the tumor bed	Yes	133	46.5	34	58.6	99	43.4	0.04
	No	153	53.5	24	41.4	129	56.6	–
Radiotherapy technique	TB-IMRT	167	58.4	33	56.9	134	58.8	0.80
	3DCRT	119	41.6	25	43.1	94	41.2	–
Fractionation regimen	CF	146	51.0	39	67.2	107	46.9	0.01
	HF	140	49.0	19	32.8	121	53.1	–
Number of fractions	≤15	86	30.1	11	19.0	75	32.9	0.01
	16–20	45	15.7	8	13.8	37	16.2	–
	21–28	70	24.5	11	19.0	59	25.9	–
	≥29	85	29.7	28	48.3	57	25.0	–
Chemotherapy	Adjuvant	211	73.8	41	70.7	170	74.6	0.58
	Neo-adjuvant	61	21.3	15	25.9	46	20.2	–
	No chemotherapy	14	4.9	2	3.4	12	5.3	–
Hormone therapy	Yes	210	73.4	44	75.9	166	72.8	0.64
	No	76	26.6	14	24.1	62	27.2	–
Fatigue (grade CTCAE v4.0)	0	101	35.3	24	41.4	77	33.8	0.51
	1	172	60.1	31	53.4	141	61.8	–
	2	13	4.5	3	5.2	10	4.4	–
Dermatitis radiation (grade CTCAE v4.0)	0	11	3.8	2	3.4	9	3.9	0.37
	1	228	79.7	42	72.4	186	81.6	–
	2	44	15.4	13	22.4	31	13.6	–
	3	3	1.0	1	1.7	2	0.9	–
Dysphagia (grade CTCAE v4.0)	0	207	72.4	47	81.0	160	70.2	0.26
	1	72	25.2	10	17.2	62	27.2	–
	2	7	2.4	1	1.7	6	2.6	–
Esophagitis (grade CTCAE v4.0)	0	265	92.7	51	87.9	214	93.9	0.21
	1	20	7.0	7	12.1	13	5.7	–
	2	1	0.3	0	0.0	1	0.4	–
Cough (grade CTCAE v4.0)	0	266	93.0	54	93.1	212	93.0	0.65
	1	17	5.9	4	6.9	13	5.7	–
	2	3	1.0	0	0.0	3	1.3	–
Dyspnea (grade CTCAE v4.0)	0	277	96.9	56	96.6	221	96.9	0.83
	1	8	2.8	2	3.4	6	2.6	–
	2	1	0.3	0	0.0	1	0.4	–

*p *Values using chi-square testing to compare patient subgroups with and without treatment interruptions, except as indicated

*DCIS* ductal carcinoma in situ, *ER* estrogen receptor, *PR* progesterone receptor, *TB-IMRT* tangential beam intensity-modulated radiotherapy, *3DCRT* three-dimensional conformal radiotherapy, *CF* conventional fractionation, *HF* hypofractionation, *CTCAE* Common Terminology Criteria for Adverse Events

^a^Unpaired Student’s *t* test

On univariate analysis, three of 23 tested factors were significantly associated with a higher risk of treatment interruptions (Table 2). All three factors were related to longer treatment courses (CF, number of radiotherapy fractions ≥29, boost to the tumor bed). In total, 19 of 140 (13.6%) patients treated with HF and 39 of 146 (26.7%) treated with CF had treatment interruptions. On multivariate analysis, the only remaining independent significant prognostic factor was the fractionation regimen: CF vs. HF; *p* = 0.007; odds ratio (95% confidence interval) 2.3 (1.3, 4.2).

**Table 2:** Univariate logistic regression results for associations with treatment interruptions

Characteristics		Odds ratio	Lower 95%CI	Upper 95%CI	*p*
Country of origin	Middle East	Reference			
	Asia	1.33	0.43	4.08	0.62
	Africa	1.27	0.53	3.05	0.60
	Europe/USA	0.30	0.09	1.04	0.06
Age at diagnosis (years)	≤Mean	Reference			
	>Mean	0.92	0.51	1.64	0.77
Body mass index	<25	Reference			
	25–29	1.29	0.55	3.03	0.57
	≥30	1.51	0.70	3.25	0.29
Menopausal status	Premenopausal	Reference			
	Postmenopausal	1.19	0.67	2.12	0.56
Marital status	Married	Reference			
	Single	0.25	0.03	1.93	0.18
Financial status	Medical insurance	Reference			
	Cash payer	0.77	0.43	1.36	0.36
Commuting distance to treatment facility (km)	≤50	Reference			
	51–100	1.04	0.46	2.36	0.93
	>100	1.46	0.54	3.99	0.46
Planning target volume (PTV)	Chest wall	Reference			
	Whole breast	0.74	0.42	1.33	0.31
Volume of PTV (cm^3^)	≤652	Reference			
	653–872	0.47	0.19	1.14	0.09
	873–1235	0.50	0.20	1.22	0.13
	≥1236	0.51	0.21	1.24	0.14
Locoregional lymph nodes treated as part of plan	Yes	Reference			
	No	0.64	0.36	1.14	0.13
Boost to the tumor bed	Yes	Reference			
	No	1.85	1.03	3.31	0.04
Radiotherapy technique	IMRT	Reference			
	3DCRT	0.93	0.52	1.66	0.80
Fractionation regimen	CF	Reference			
	HF	2.32	1.27	4.26	<0.01
Number of radiotherapy fractions	≤15	Reference			
	16–20	0.68	0.25	1.83	0.44
	21–28	0.79	0.32	1.94	0.60
	≥29	0.30	0.14	0.65	<0.01
Chemotherapy	Adjuvant	Reference			
	Neo-adjuvant	0.74	0.38	1.45	0.38
	No chemotherapy	1.45	0.31	6.72	0.64
Hormone therapy	Yes	Reference			
	No	1.25	0.64	2.44	0.51
Fatigue (grade CTCAE v4.0)	0	Reference			
	>0	0.74	0.41	1.33	0.31
Dermatitis radiation (grade CTCAE v4.0)	0	Reference			
	>0	1.15	0.24	5.48	0.86
Dysphagia (grade CTCAE v4.0)	0	Reference			
	>0	0.55	0.27	1.13	0.10
Esophagitis (grade CTCAE v4.0)	0	Reference			
	>0	2.10	0.81	5.46	0.13
Cough (grade CTCAE v4.0)	0	Reference			
	>0	0.98	0.32	3.06	0.97
Dyspnea (grade CTCAE v4.0)	0	Reference			
	>0	1.13	0.23	5.58	0.88
Any acute radiation reaction (grade CTCAE v4.0)	0, 1	Reference			
	2, 3	1.69	0.87	3.28	0.12

*CI* confidence interval, *IMRT* intensity-modulated radiotherapy, *3DCRT* three-dimensional conformal radiotherapy, *CF* conventional fractionation, *HF* hypofractionation, *CTCAE* Common Terminology Criteria for Adverse Events

Concerning the reasons for treatment interruptions, no statistically significant differences were detected between the patients treated with CF and HF (Table 3). However, treatment interruptions were on average longer for patients treated with CF (3.2 days vs. 2.3 days; *p* = 0.02; Table 3).

**Table 3:** Reason for and length of treatment interruptions stratified by fractionation regimen

Reason for treatment interruptions		Total		Fractionation regimen				*p*
				HF		CF		
		*n*	%	*n*	%	*n*	%	
Public holidays	Yes	33	56.9	11	57.9	22	56.4	0.91
	No	25	43.1	8	42.1	17	43.6	–
	Days, mean (SD)	1.9	(1.8)	1.6	(1.5)	2.1	(2.0)	0.34^a^
Patient unwillingness	Yes	23	39.7	8	42.1	15	38.5	0.79
	No	35	60.3	11	57.9	24	61.5	–
	Days, mean (SD)	0.6	(0.9)	0.5	(0.7)	0.6	(0.9)	0.80^a^
Machine breakdown	Yes	8	13.8	1	5.3	7	17.9	0.19
	No	50	86.2	18	94.7	32	82.1	–
	Days, mean (SD)	0.3	(0.8)	0.1	(0.2)	0.4	(1.0)	0.16^a^
Radiation reactions	Yes	4	6.9	0	0	4	10.3	0.15
	No	54	93.1	19	100	35	89.7	–
	Days, mean (SD)	0.1	(0.3)	0	(0)	0.1	(0.3)	0.16^a^
Unspecified	Yes	1	1.7	1	5.3	0	0	0.15
	No	57	98.3	18	94.7	39	100	–
	Days, mean (SD)	0.1	(0.4)	0.2	(0.7)	0	(0)	0.15^a^
Treatment interruptions	Yes	58	20.3	19	13.6	39	26.7	0.01
	No	228	79.7	121	86.4	107	73.3	–
	For one reason	47	81.0	17	89.5	30	76.9	0.25
	For two reasons	11	19.0	2	10.5	9	23.1	–
	Days, mean (SD)	2.9	(1.4)	2.3	(1.2)	3.2	(1.4)	0.02^a^
Prolongation of the prescribed overall treatment time after compensation for treatment interruptions	0 days	24	41.4	9	47.4	15	38.5	0.68
	1 day	15	25.9	6	31.6	9	23.1	–
	2 days	9	15.5	1	5.3	8	20.5	–
	3 days	5	8.6	1	5.3	4	10.3	–
	4 days	3	5.2	1	5.3	2	5.1	–
	5 days	2	3.4	1	5.3	1	2.6	–

*p *Values using chi-square testing to compare patient subgroups treated with HF or CF, except as indicated

*CF* conventional fractionation, *HF* hypofractionation

^a^Unpaired Student’s *t* test

In accordance with our departmental policy, treatment interruptions were compensated by treating the corresponding patients on weekends within the prescribed overall treatment time. After compensation for treatment interruptions, eventually 41.4% of the patients with treatment interruptions completed their treatment within the prescribed overall treatment time, corresponding to 88.1% of the total study population. The remaining patients experienced a prolongation of the prescribed overall treatment time of 1–5 days (Table 3).

## Discussion

Our study shows that a significant proportion of our patients experienced unintended treatment interruptions (20.3%). The compliance to the prescribed radiotherapy schedule was significantly better with HF than with CF (patients with treatment interruptions; 13.6% vs. 26.7%). The data suggest that the better compliance was basically related to the shorter overall treatment time of HF (3–4 weeks) compared with CF (5–6.5 weeks).

Several randomized trials have shown that HF is equally effective in long-term disease control and late radiation effects compared with CF in adjuvant breast cancer radiotherapy [20–23]. The main motivation for developing protracted radiotherapy regimens was the benefit to patients and health services in terms of convenience and cost. Recent breast cancer studies suggested that HF is also associated with a significantly lower acute skin reaction rate compared with CF [7, 24, 25]. Our study revealed another advantage of HF over CF: a significantly better patient compliance with the prescribed radiotherapy schedule.

Noncompliance with the prescribed radiotherapy schedule can have multiple deleterious effects. For postoperative radiotherapy of breast cancer, a prolongation of the overall treatment time of more than 1 week has been shown to decrease the 5‑year local control rate by 5% [26]. The management of the increased number of recurrences may place additional burden on the health-care system. Disturbances in the clinical workflow by noncompliant (“no-show”) patients occupying treatment slots on the linear accelerator may indirectly cause treatment delays for other patients and an extension of the work day. Compensation of missed radiotherapy fractions during the working week by additional treatment on weekends will further increase costs in terms of time and effort.

In a large study of 2184 patients receiving radiotherapy with curative intent for various malignancies in an American urban academic cancer center, 20.2% missed multiple radiotherapy fractions, 17.4% a single radiotherapy fraction, and 62.4% no radiotherapy fractions. The median number of missed treatments was 3. Similar to our study, the statistical analysis identified “prescribed longer radiotherapy courses” as a statistically significant independent predictor of noncompliance. The authors suggested that this finding may provide additional rationale for adopting shortened radiotherapy schedules as a means of improving patient adherence to prescribed therapy [27]. Other predictors for noncompliance identified in the previously cited study were “particular cancer diagnoses,” “low socioeconomic status,” and “treatment during winter months.” “Distance from the patients’ home to the radiotherapy facility” [28–30] and “patients from households that lost family income” [31] have been reported as predictors of noncompliance with the prescribed radiotherapy schedule by other study groups. It is likely that factors influencing compliance depend to a significant extent on individual circumstances like the location of the radiotherapy facility, infrastructure of the region, and socioeconomic status of the population, and may therefore vary between treatment facilities. However, in our study CF, which was the longer radiotherapy schedule compared with HF, was the only significant predictor of noncompliance on multivariate analysis of 23 factors.

The limitations of our study should be noted. Owing to the relatively limited patient number (*n* = 286), possible influencing factors may not have reached statistical significance. The socioeconomic and psycho-oncological status of the patients could not be evaluated because of lack of data. Moreover, owing to the retrospective nature of the study, a selection bias of patients treated with HF and CF cannot be excluded with certainty.

Despite all efforts to avoid a prolongation of the prescribed overall treatment time by thorough education of the patient and compensation of missed radiotherapy fractions by treatment on weekends, 34 of 286 patients (11.9%) in our study eventually experienced a moderate prolongation of the prescribed overall treatment time of 1–5 days. Data concerning the detrimental effect of treatment interruptions in adjuvant breast cancer radiotherapy are scarce. However, a significant decrease in the 5‑year local control rate after treatment interruptions of more than 1 week has been reported [26].

## Conclusion

A significant proportion of breast cancer patients in our study experienced treatment interruptions. Compliance with the prescribed radiotherapy schedule was significantly better for patients treated with HF than for those treated with CF. The data suggest that the better compliance is basically related to the shorter overall treatment time of HF (3–4 weeks) compared with CF (5–6.5 weeks). This finding may add to the treatment decision in favor of HF in particular in situations with expected lower compliance with longer radiotherapy schedules.

## References

[1] Thames HD, Kuban D, Levy LB, Horwitz EM, Kupelian P, Martinez A, Michalski J, Pisansky T, Sandler H, Shipley W, Zelefsky M, Zietman A (2010). The role of overall treatment time in the outcome of radiotherapy of prostate cancer: an analysis of biochemical failure in 4839 men treated between 1987 and 1995. Radiotherapy and oncology : journal of the European Society for Therapeutic. Radiol Oncol.

[2] Perez CA, Grigsby PW, Castro-Vita H, Lockett MA (1995). Carcinoma of the uterine cervix. I. Impact of prolongation of overall treatment time and timing of brachytherapy on outcome of radiation therapy. Int. J. Radiat. Oncol. Biol. Phys..

[3] Fowler JF, Lindstrom MJ (1992). Loss of local control with prolongation in radiotherapy. Int. J. Radiat. Oncol. Biol. Phys..

[4] Ohri N, Rapkin BD, Guha C, Kalnicki S, Garg M (2016). Radiation therapy noncompliance and clinical outcomes in an urban academic cancer center. Int. J. Radiat. Oncol. Biol. Phys..

[5] Bese NS, Hendry J, Jeremic B (2007). Effects of prolongation of overall treatment time due to unplanned interruptions during radiotherapy of different tumor sites and practical methods for compensation. Int J Radiat Oncol Biol Phys.

[6] Withers HR, Taylor JM, Maciejewski B (1988). The hazard of accelerated tumor clonogen repopulation during radiotherapy. Acta Oncol.

[7] Rudat V, Nour A, Ghaida SA, Alaradi A (2016). Impact of hypofractionation and tangential beam IMRT on the acute skin reaction in adjuvant breast cancer radiotherapy. Radiat Oncol.

[8] Rudat V, Nour A, Alaradi AA, Mohamed A, Altuwaijri S (2014). In vivo surface dose measurement using GafChromic film dosimetry in breast cancer radiotherapy: comparison of 7‑field IMRT, tangential IMRT and tangential 3D-CRT. Radiat Oncol.

[9] Rudat V, Alaradi AA, Mohamed A, Ai-Yahya K, Altuwaijri S (2011). Tangential beam IMRT versus tangential beam 3D-CRT of the chest wall in postmastectomy breast cancer patients: a dosimetric comparison. Radiat Oncol.

[10] RTOG (2016). Breast Cancer Atlas.

[11] Rudat V, Hammoud M, Pillay Y, Alaradi AA, Mohamed A, Altuwaijri S (2011). Impact of the frequency of online verifications on the patient set-up accuracy and set-up margins. Radiat Oncol.

[12] Becker-Schiebe M, Stockhammer M, Hoffmann W, Wetzel F, Franz H (2016). Does mean heart dose sufficiently reflect coronary artery exposure in left-sided breast cancer radiotherapy?: Influence of respiratory gating. Strahlenther Onkol.

[13] Hepp R, Ammerpohl M, Morgenstern C, Nielinger L, Erichsen P, Abdallah A, Galalae R (2015). Deep inspiration breath-hold (DIBH) radiotherapy in left-sided breast cancer: Dosimetrical comparison and clinical feasibility in 20 patients. Strahlenther Onkol.

[14] Schonecker S, Heinz C, Sohn M, Haimerl W, Corradini S, Pazos M, Belka C, Scheithauer H (2016). Reduction of cardiac and coronary artery doses in irradiation of left-sided breast cancer during inspiration breath hold : A planning study. Strahlenther Onkol.

[15] Bahrainy M, Kretschmer M, Jost V, Kasch A, Wurschmidt F, Dahle J, Lorenzen J (2016). Treatment of breast cancer with simultaneous integrated boost in hybrid plan technique : Influence of flattening filter-free beams. Strahlenther Onkol.

[16] Jost V, Kretschmer M, Sabatino M, Wurschmidt F, Dahle J, Ueberle F, Lorenzen J (2015). Heart dose reduction in breast cancer treatment with simultaneous integrated boost: Comparison of treatment planning and dosimetry for a novel hybrid technique and 3D-CRT. Strahlenther Onkol.

[17] Ott OJ, Strnad V, Stillkrieg W, Uter W, Beckmann MW, Fietkau R (2017). Accelerated partial breast irradiation with external beam radiotherapy : First results of the German phase 2 trial. Strahlenther Onkol.

[18] Lohr F, El-Haddad M, Dobler B, Grau R, Wertz HJ, Kraus-Tiefenbacher U, Steil V, Madyan YA, Wenz F (2009). Potential effect of robust and simple IMRT approach for left-sided breast cancer on cardiac mortality. Int. J. Radiat. Oncol. Biol. Phys..

[19] Rudat V, Brune-Erbe I, Noureldin A, Bushnag Z, Almuraikhi N, Altuwaijri S (2012). Epidemiology of breast cancer patients at a tertiary care center in the Eastern Province of Saudi Arabia. Gulf J Oncolog.

[20] Yarnold J, Ashton A, Bliss J, Homewood J, Harper C, Hanson J, Haviland J, Bentzen S, Owen R (2005). Fractionation sensitivity and dose response of late adverse effects in the breast after radiotherapy for early breast cancer: long-term results of a randomised trial. Radiother Oncol.

[21] Owen JR, Ashton A, Bliss JM, Homewood J, Harper C, Hanson J, Haviland J, Bentzen SM, Yarnold JR (2006). Effect of radiotherapy fraction size on tumour control in patients with early-stage breast cancer after local tumour excision: long-term results of a randomised trial. Lancet Oncol.

[22] Whelan TJ, Pignol JP, Levine MN, Julian JA, MacKenzie R, Parpia S, Shelley W, Grimard L, Bowen J, Lukka H, Perera F, Fyles A, Schneider K, Gulavita S, Freeman C (2010). Long-term results of hypofractionated radiation therapy for breast cancer. N. Engl. J. Med..

[23] Haviland JS, Owen JR, Dewar JA, Agrawal RK, Barrett J, Barrett-Lee PJ, Dobbs HJ, Hopwood P, Lawton PA, Magee BJ, Mills J, Simmons S, Sydenham MA, Venables K, Bliss JM, Yarnold JR (2013). The UK Standardisation of Breast Radiotherapy (START) trials of radiotherapy hypofractionation for treatment of early breast cancer: 10-year follow-up results of two randomised controlled trials. Lancet Oncol.

[24] Shaitelman SF, Schlembach PJ, Arzu I, Ballo M, Bloom ES, Buchholz D, Chronowski GM, Dvorak T, Grade E, Hoffman KE, Kelly P, Ludwig M, Perkins GH, Reed V, Shah S, Stauder MC, Strom EA, Tereffe W, Woodward WA, Ensor J, Baumann D, Thompson AM, Amaya D, Davis T, Guerra W, Hamblin L, Hortobagyi G, Hunt KK, Buchholz TA, Smith BD (2015). Acute and short-term toxic effects of conventionally fractionated vs hypofractionated whole-breast irradiation: a randomized clinical trial. JAMA Oncol.

[25] Jagsi R, Griffith KA, Boike TP, Walker E, Nurushev T, Grills IS, Moran JM, Feng M, Hayman J, Pierce LJ (2015). Differences in the acute toxic effects of breast radiotherapy by fractionation schedule: comparative analysis of physician-assessed and patient-reported outcomes in a large multicenter cohort. JAMA Oncol.

[26] Bese NS, Sut PA, Ober A (2005). The effect of treatment interruptions in the postoperative irradiation of breast cancer. Oncology.

[27] Ohri N, Rapkin BD, Guha D, Haynes-Lewis H, Guha C, Kalnicki S, Garg M (2015). Predictors of radiation therapy noncompliance in an urban academic cancer center. Int. J. Radiat. Oncol. Biol. Phys..

[28] Meden T, St. John-Larkin C, Hermes D, Sommerschield S (2002). Relationship between travel distance and utilization of breast cancer treatment in rural northern Michigan. JAMA.

[29] Nattinger AB, Kneusel RT, Hoffmann RG, Gilligan MA (2001). Relationship of distance from a radiotherapy facility and initial breast cancer treatment. J. Natl. Cancer Inst..

[30] Athas WF, Adams-Cameron M, Hunt WC, Amir-Fazli A, Key CR (2000). Travel distance to radiation therapy and receipt of radiotherapy following breast-conserving surgery. J. Natl. Cancer Inst..

[31] Arrossi S, Matos E, Zengarini N, Roth B, Sankaranayananan R, Parkin M (2007). The socio-economic impact of cervical cancer on patients and their families in Argentina, and its influence on radiotherapy compliance. Results from a cross-sectional study. Gynecol. Oncol..

